# LINC00184 plays an oncogenic role in non‐small cell lung cancer via regulation of the miR‐524‐5p/HMGB2 axis

**DOI:** 10.1111/jcmm.16247

**Published:** 2021-10-15

**Authors:** Wuming Wang, Lin Li, Long Zhao

**Affiliations:** ^1^ Department of Thoracic Surgery Jiangxi Provincial Chest Hospital Nanchang China; ^2^ Department of Thoracic Surgery Ji'an Central People's Hospital Ji'an China

**Keywords:** HMGB2, LINC00184, miR‐524‐5p, non‐small cell lung cancer, proliferation

## Abstract

Non‐small cell lung cancer (NSCLC) is the most common type of lung cancer. We aimed to investigate the role of LINC00184 in NSCLC. Migration, proliferation and invasion of NSCLC cells were analysed using the wound healing assay, cell counting kit‐8 assay and transwell assay, respectively. Apoptosis and cell cycle were assessed using flow cytometry. Online bioinformatics tools were utilized to predict downstream microRNAs (miRNA) or genes related to LINC00184 expression. The RNA pull‐down experiment and luciferase reporter assay were performed to verify the predictions thereof. LINC00184, miR‐524‐5p, and high mobility group 2 protein (HMGB2) expression levels in NSCLC tissues and cell lines were detected using quantitative real‐time polymerase chain reaction. An NSCLC mouse model was constructed for in vivo experiments. LINC00184 overexpression was observed in NSCLC tissues and cell lines and was found to be correlated with poor prognosis. LINC00184 knockdown inhibited cell proliferation, migration and invasion, induced cell cycle arrest and accelerated apoptosis in NSCLC cell lines. LINC00184 suppressed tumour growth and proliferation in NSCLC mouse models and directly targeted the miR‐524‐5p/HMGB2 axis. Moreover, the expression levels of LINC00184 and HMGB2 were negatively correlated with miR‐524‐5p expression, whereas LINC00184 expression was positively correlated with HMGB2 expression. LINC00184 affected the cell cycle, proliferation, apoptosis, migration and invasion in NSCLC via regulation of the miR‐524‐5p/HMGB2 axis.

## 1 INTRODUCTION

Despite tremendous advances in medical technology, the incidence of lung cancer (LC) is the highest among malignant tumours; LC also remains the leading cause of cancer‐related deaths in the world.^1^ According to GLOBOCAN 2018, there were nearly 2.1 million new cases of LC and 1.8 million deaths worldwide in 2018.^2^ In China, there were 733 000 new cases of LC and 610 000 deaths in 2015.^3^ LC is classified by pathological type; 80%–85% of these cases are non‐small cell lung cancer (NSCLC).^4^ The majority of NSCLC patients are diagnosed in the latter stages and miss the opportunity for surgery.^5^ With the current treatment strategies, the 5‐year survival rate of NSCLC is 16%. Dual‐drug chemotherapy combined with platinum is the first choice for treatment of advanced NSCLC. Its efficacy is approximately 30%–40%, with a median survival time of 8‐10 months.

The pathogenesis of NSCLC is a complicated process with multiple steps and factors.

It is well known that smoking is one of the major risk factors for an increase in NSCLC.^6^ Other factors associated with increased NSCLC risk include secondhand smoke, family history, human immunodeficiency virus infection, occupational exposure to carcinogens, radiation exposure and air pollution.^7^ Traditional treatments for NSCLC include surgery, radiotherapy, chemotherapy and immunotherapy. If NSCLC is diagnosed at an early stage, prognosis during surgical resection is good.^8^ Therefore, early detection of NSCLC has become important in clinical diagnosis and treatment. NSCLC is related to disorders of various cell processes, such as cell cycle progression, proliferation, apoptosis and angiogenesis. In recent years, the emergence of molecular targeted drugs has been a major advancement in NSCLC treatment.^9^ For early screening, diagnosis, treatment and prognosis, it is important to thoroughly clarify the molecular biological mechanism underlying the occurrence and development of NSCLC.

In recent years, a variety of biomarkers have been shown to be associated with the occurrence and development of NSCLC, such as PD‐L1,^10^ TMPRSS4,^11^ and Ect2.^12^ Long non‐coding RNAs (lncRNAs), previously known as ‘transcription noise’, are closely related to a number of cancers. lncRNAs have been reported to regulate the occurrence and development of cancer and play a role similar to that of an oncogene. lncRNAs were confirmed to affect the sensitivity of cancer radiotherapy. According to previous studies, multiple lncRNAs including MALAT1,^13^ HNF1A‐AS1,^14^ and MAFG‐AS1^15^ play a role in NSCLC tumour progression. We found that lncRNA LINC00184 was highly expressed in LC tumour tissues based on The Cancer Genome Atlas (TCGA) database. At present, there are few studies on the role of LINC00184 in tumours. However, LINC00184 was reported to be highly expressed in oesophageal cancer and regulate glycolysis and mitochondrial oxidative phosphorylation.^16^ Nevertheless, the functional role of LINC00184 in tumours requires clarification. Therefore, we designed the current study to investigate the function and underlying mechanism of action of LINC00184 in NSCLC.

In the present study, we observed LINC00184 up‐regulation in NSCLC tumours, which was correlated with poor prognosis. Additionally, we found that LINC00184 was overexpressed in NSCLC cell lines. Down‐regulation of LINC00184 suppressed cell proliferation, migration, and invasion and enhanced apoptosis by targeting microRNA‐524‐5p (miR‐524‐5p)/high mobility group 2 protein (HMGB2) in vitro. Additionally, we confirmed that LINC00184 knockdown repressed tumour growth and proliferation in vivo.

## 2 MATERIALS AND METHODS

### 2.1 TCGA database

The LINC00184 expression profile of 348 cases of LC tumour tissues and 46 cases of normal adjacent tissues was downloaded from TCGA database (http://cancergenome.nih.gov/). The statistical analysis was then performed using SPSS 20.0 (IBM, USA).

### 2.2 Tissue sample collection

NSCLC tumour tissues (n = 98) and matched adjacent noncancerous tissues (n = 98) were harvested from NSCLC patients subjected to radical resection in Jiangxi Provincial Chest Hospital from April 2014 to January 2018. All NSCLC tumour tissues were pathologically confirmed and staged according to the standard of International Union for Cancer Control (UICC) tumour, node and metastasis (TNM) staging system.^17^ The expression of LINC00184 was detected in 98 cases of NSCLC tumour tissues and median expression served as a limit. The 98 cases of tumour tissues in our research were grouped into the high and low LINC00184 expression groups in accordance with LINC00184 median expression. The time from surgery to death of these patients was recorded and represented as an overall survival rate. None of the NSCLC patients underwent pre‐operative antitumour treatment. This study was approved by the Ethics Committee of Jiangxi Provincial Chest Hospital and carried out in accordance with the Guidelines of Jiangxi Provincial Chest Hospital; the study adhered to the ethical guidelines of the 1975 Declaration of Helsinki. Written informed consents were acquired from patients before the study.

### 2.3 Cell culture

A normal human bronchial epithelial cell line (16HBE) and NSCLC cell lines (NCI‐H1359, H1650, A549, H1975 and HCC827) were purchased from the Cell Bank of the Chinese Academy of Science (Shanghai, China). NCI‐H1359, A549, H1975 and 16HBE cell lines were maintained in Dulbecco's modified Eagle's Medium (DMEM, Gibco, MA, USA) supplemented with 10% foetal bovine serum (FBS, v/v, Invitrogen, Carlsbad, CA, USA), 100 μg/mL streptomycin (Invitrogen) and 100 μg/mL penicillin (Invitrogen) and maintained at 37°C and 5% CO_2_. H1650 and HCC827 cell lines were cultured in Roswell Park Memorial Institute (RPMI) 1640 medium (Gibco) supplemented with 10% FBS at 37°C and 5% CO_2_.

### 2.4 Cell transfection

Small interfering LINC00184‐1 (siLINC00184‐1) and siLINC00184‐2, small interfering negative control vector (siNC), miR‐524‐5p NC/miR‐524‐5p mimic, miR‐1305 NC/miR‐1305 mimic, miR‐202‐5p NC/miR‐202‐5p mimic, LINC00184/NC, siHMGB2, short hairpin LINC00184 (shLINC00184) and shNC were all purchased from RiboBio (Guangzhou, China). Cells were pre‐seeded in 12‐well plates and cultured until 70% confluence. The experimental cell lines were then transfected using Lipofectamine 2000 (Invitrogen).

### 2.5 Quantitative real‐time PCR (qRT‐PCR)

The total RNA in NSCLC tissue samples and cell lines was extracted using Trizol reagent obtained from Thermo Fisher Scientific. PrimeScript RT reagent purchased from Takara (Tokyo, Japan) was used to synthesize cDNA. An ABI 7500 fast real‐time PCR system (Thermo Fisher Scientific) was used to conduct qRT‐PCR under the following reaction conditions: 95°C for 5 minutes, 39 cycles of 95°C for 15 seconds, 60°C for 30 seconds and 72°C for 30 seconds. The expression levels of LINC00184 and HMGB2 were determined according to the 2^−ΔΔCt^ method with GAPDH as an internal control. Similarly, the expression levels of miR‐524‐5p, miR‐1305, and miR‐202‐5p were determined with U6 as an internal control. The primer sequences were as follows: LINC00184: 5′‐ATGGCTCTCCTTTCCCA‐3′, 5′‐TGATGCCTTGCTTGACC‐3′; miR‐524‐5p: 5′‐GGGCTACAAAGGGAAGCAC‐3′, 5′‐CACCACCAACCACCACTAAT‐3′; miR‐1305:5′‐AGCGCTTTTCAACTCTAATGG‐3′, 5′‐TCCTCCTCTCCTTCCTTCTC‐3′; miR‐202‐5p: 5′‐AGCGCTTCCTATGCATATACT‐3′, 5′‐GTTGTGGTTGGTTGGTTTGT‐3′; HMGB2: 5′‐GCCAACAGGCTCAAAGAA‐3′, 5′‐CACACATTCCACACGCA‐3′; GAPDH: 5′‐ATCACCATCTTCCAGGAGCGA‐3′, 5’‐ATGGCATGGACTGTGGTCAT‐3’; and U6: 5′‐AACGAGACGACGACAGAC‐3′, 5’‐GCAAATTCGTGAAGCGTTCCATA‐3’.

### 2.6 Western blotting

A pre‐cooled RIPA lysis buffer (Sigma) was applied to lyse the experimental cell lines. A bicinchoninic acid protein (BCA) assay kit (Sigma) was used to detect the total protein concentration. SDS‐PAGE loading buffer (5X) was mixed with 10 µg total proteins and heated at 100°C for 5 minutes. Thereafter, SDS‐PAGE (4%‐12%) was used to separate proteins. The proteins were subsequently transferred to PVDF membranes (0.45 µm, EMD Millipore) and placed in 4°C for 2 hours. Next, 5% non‐fat milk was used to block the PVDF membranes at room temperature for 1 hour. After incubation with primary antibodies at 4°C overnight, the PVDF membranes were treated with horseradish peroxidase (HRP)‐conjugated secondary antibodies (1:20 000; Southern Biotech) placed in 37°C for 1 hour. Immobilon Western Chemiluminescent HRP substrate (EMD Millipore) was used to visualize the protein bands. Primary antibodies (Abcam) were as follows: anti‐HMGB2 (1:500, ab67282), anti‐cleaved caspase‐3 (1:500, ab2302), anti‐p53 (1:500, ab32389) and anti‐β‐actin (1:1000, ab8227).

### 2.7 Immunohistochemistry (IHC) staining

Paraffin‐embedded tissue was sectioned (3.5 μm thickness) and maintained at 60°C for 2 hours. Sections were dewaxed with xylene and rehydrated with a graded ethanol series. The endogenous peroxidase activity was blocked by H_2_O_2_. Thereafter, primary antibodies (anti‐HMGB2 and anti‐Ki67) were added to the tumour sections and incubated overnight at 4°C. Secondary antibodies were subsequently added and the sections were maintained at room temperature for 30 minutes. A DAB kit (DAB‐1031; MXB Biotechnology, China) was used to stain the sections. Haematoxylin was applied to counterstain sections for 3 minutes before observation under a microscope.

### 2.8 Cell Counting Kit‐8 (CCK‐8) assay

Cell lines (5 × 10^3^ cells/plate) were seeded in a 96‐well plate at 37°C and 5% CO_2_. After 12, 24, 48 and 72 hours of incubation, 10 μL CCK‐8 solution (Solarbio, Beijing, China), was added to each well. Cell lines were continuously cultured for another 2 hours at 37°C. The optical density of each sample was measured at a wavelength of 450 nm using a microplate reader (Thermo Fisher Scientific, Waltham, MA, USA).

### 2.9 Flow cytometry

A flow cytometer (BD Biosciences, Franklin Lakes, NJ, USA) was used to detect apoptosis and stages of the cell cycle in the experimental group. Transfected cell lines were collected after centrifugation and supplemented with 1X binding buffer (Invitrogen). To visualize apoptotic cells, propidium iodide (PI) and FITC‐Annexin V were applied. To elucidate the cell cycle stages, cell lines were stained with PI and RNase (Becton Dickinson, 550825, San Diego, CA, USA) for 30 minutes in the dark. A FL2 band‐pass filter was employed to collect the fluorescent emissions.

### 2.10 Wound healing assay

After transfection for 48 hours, cells (2.5 × 10^5^ cells/well) were implanted onto 12‐well plates overnight. The monolayer was gently scratched using a pipette tip (10 μL). Thereafter, the wells were washed twice with PBS and fresh medium was added, followed by incubation for 24 hours. The gap closures were captured using a microscope (Olympus, Tokyo, Japan).

### 2.11 Transwell assay

Transwell chambers, (Costar, Cambridge, MA, USA), were pre‐coated with Matrigel (BD Biosciences, San Jose, CA, USA) and used to measure cell invasion. Briefly, Matrigel (100 µL, 1:7 dilution) was added into each chamber and maintained at 37°C for 2 hours. The cells incubated in serum‐free medium were subsequently supplemented into the upper chamber at a density of 12 × 10^4^ cells/chamber. Thereafter, a total of 0.6 mL medium, including 20% FBS, was added into the lower chamber and served as a chemoattractant. After 24 hours, 4% paraformaldehyde was applied to fix the filters for 20 minutes and haematoxylin was used as a stain for 10 minutes. A cotton swab was used to remove the non‐invading cell lines present on the upper surface. The number of invading cells in five random fields was counted under a microscope (Olympus).

### 2.12 Luciferase reporter assay

The target microRNA (miRNA) of LINC00184 and the target gene thereof were predicted using miRDB and TargetScan, respectively. Wild‐type (Wt) and mutant (Mut) luciferase reporter vectors of LINC00184 containing miR‐524‐5p, miR‐1305 and miR‐202‐5p binding sites were designed and constructed by GenePharma (Shanghai, China). Additionally, HMGB2‐Mut and HMGB2‐WT vectors were obtained from GenePharma. LINC00184‐WT or LINC00184‐Mut vectors were transfected into HEK293T cells and cotransfected with miR‐524‐5p mimic/miR‐524‐5p NC, miR‐1305 mimic/miR‐1305 NC or miR‐202‐5p mimic/miR‐202‐5p NC with Lipofectamine 2000. Furthermore, HMGB2‐Mut or HMGB2‐WT were cotransfected with miR‐524‐5p mimic/miR‐NC in HEK293T cells. After 48 hours of transfection, the relative luciferase activity of experimental cells was measured by a dual‐luciferase reporter assay system (Promega, Madison, WI, USA).

### 2.13 RNA pull‐down assay

The LINC00184 biotinylated DNA and negative control probes (Invitrogen) were dissolved in binding buffer (500 μL). Streptavidin‐coated magnetic beads (Sigma) were used to incubate the probes at 37°C for 3 hours, resulting in probe‐coated magnetic beads. The probe‐coated beads were applied to treat cell lysates. qRT‐PCR was used to detect the enrichment of miR‐524‐5p, miR‐1305 and miR‐202‐5p mimics.

### 2.14 Xenograft NSCLC nude mouse model

BALB/c nude mice (n = 6 mice/group, 4 weeks old, 18‐22 g) were purchased from Slac Laboratories (Shanghai, China). NSCLC A549 cells (3 × 10^5^ cell/mouse), transfected with shLINC00184 or shNC, were injected subcutaneously into BALB/c nude mice. Tumours volumes of the two groups mice were evaluated and recorded weekly. After 4 weeks, mice were killed and tumours were collected, photographed and weighted. The expression levels of HMGB2 and Ki67 in mouse tumour tissues were measured using IHC. The animal experiment was approved by Ethics Committee of Jiangxi Provincial Chest Hospital and carried out in accordance with the Guidelines for Animal Use in the National Institutes of Health.

### 2.15 Statistical analysis

All statistical analyses were performed using SPSS version 20.0 software (IBM, SPSS, Chicago, IL, USA). One‐way analysis of variance and Student's t test were performed to compare the differences between multiple or two groups, respectively. The relationship between NSCLC clinical features (including age, gender, smoking, differentiation, T classification, N classification, distant metastasis and clinical stage) and LINC00184 expression was assessed using Pearson's chi‐square tests. Kaplan‐Meier survival analysis was utilized to measure the survival rate. *P* < 0.05 was regarded as statistically significant.

## 3 RESULTS

### 3.1 LINC00184 was up‐regulated in NSCLC tissues and cell lines

**FIGURE 1:**
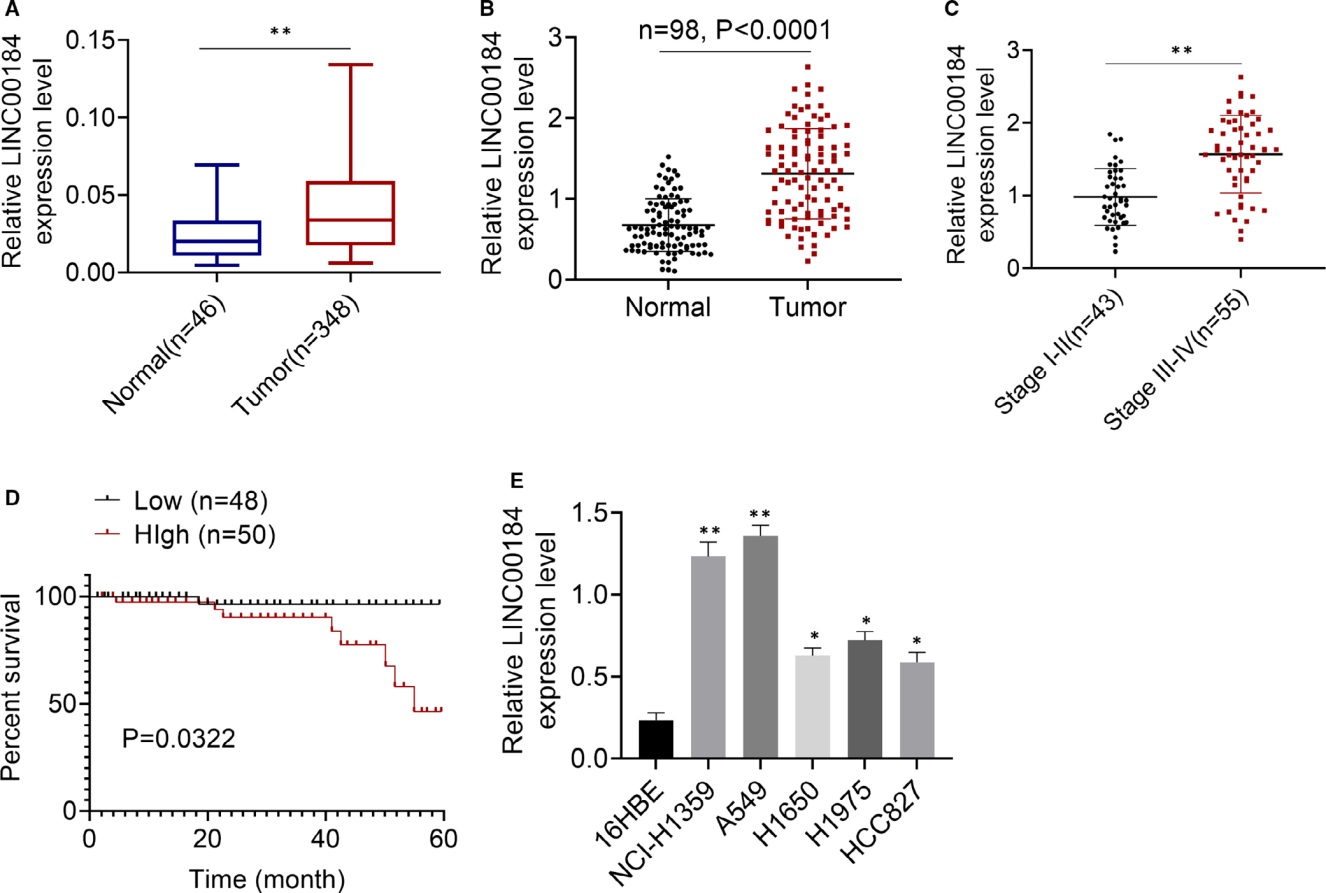
LINC00184 was highly expressed in NSCLC tumour tissues and cell lines. A, The data of LINC00184 expressions in 348 cases of NSCLC tumour tissues and 46 cases of normal tissues were downloaded from TCGA database. B, The expressions of LINC00184 in 98 cases of NSCLC tumour tissues and matched non‐tumour tissues were detected using qRT‐PCR. C, The expressions of LINC00184 in 43 cases of NSCLC stage I‐II patients tumour tissues and 55 cases of NSCLC stage III‐IV patients tumour tissues were detected using qRT‐PCR. D, Survival rates were analysed using Kaplan‐Meier survival analysis. E, The expressions of LINC00184 in human NSCLC cell lines and normal cell lines were detected using qRT‐PCR. Data are shown as mean ± SD. *P < 0.05, **P < 0.01 vs normal group, Stage I‐II group or 16HBE cell line

We downloaded LINC00184 expression level data in 348 cases of NSCLC tumour tissues and 46 cases of adjacent normal tissues from TCGA database; results showed that LINC00184 was significantly overexpressed in NSCLC tumour tissues compared to that in normal tissues (*P* < 0.01, Figure 1A). A total of 98 pairs of NSCLC tumour tissues and non‐tumour tissues were collected and the expression levels of LINC00184 were detected via qRT‐PCR. As shown in Figure 1B, the relative LINC00184 expression levels in the tumour group were significantly higher than those in the normal group (*P* < 0.0001). Additionally, the LINC00184 expression levels in the tissues of NSCLC stage III‐IV patients were notably higher than in the tissues of stage I‐II patients (*P* < 0.01, Figure 1C). The 98 cases of NSCLC tumour tissues were grouped into high and low LINC00184 expression groups according to the LINC00184 median expression level. Thereafter, the survival rate and the relationship between LINC00184 expression and NSCLC clinical features were analysed. As shown in Figure 1D, high LINC00184 expression levels indicated shorter survival rates. Furthermore, LINC00184 expression levels in NSCLC tumour tissues was notably associated with T classification, N classification and clinical stage (Table 1). Moreover, the LINC00184 expression levels in human NSCLC cell lines, including NCI‐H1359, A549, H1650, H1975 and HCC827, were detected by qRT‐PCR. Results showed that LINC00184 was considerably overexpressed in NSCLC tumour cell lines when compared to that in the 16HBE cell line (*P* < 0.05 or *P* < 0.01). The above data indicated that LINC00184 was overexpressed in NSCLC tumour tissues and cell lines and that its up‐regulation in NSCLC tumour tissues indicated a poor prognosis for NSCLC patients.

**TABLE 1:** The correlation between LINC00184 expression and NSCLC clinical pathology

Characteristics	Number of patients	LINC00184 Low expression (<median)	LINC00184 High expression (≥median)	*P* value
Number	98	48	50	
Ages (years)				
<65	45	22	23	.527
≥65	53	26	27	
Gender				
Female	46	24	22	.382
Male	52	24	28	
Smoking				
Yes	50	23	27	.163
No	48	25	23	
Differentiation				
Well differentiated	44	22	22	.448
Lowly or undifferentiated	54	26	28	
T classification				
T1 + T2	45	26	19	.017
T3 + T4	53	22	31	
N classification				
N0 + N1	46	28	18	.017
N2 + N3	52	20	32	
Distant metastasis				
M1	48	27	21	.113
M0	50	21	29	
Clinical stage				
I‐II	43	25	18	.001
III‐IV	55	23	32	

### 3.2 Knockdown of LINC00184 inhibited cellular activities in NSCLC

**FIGURE 2:**
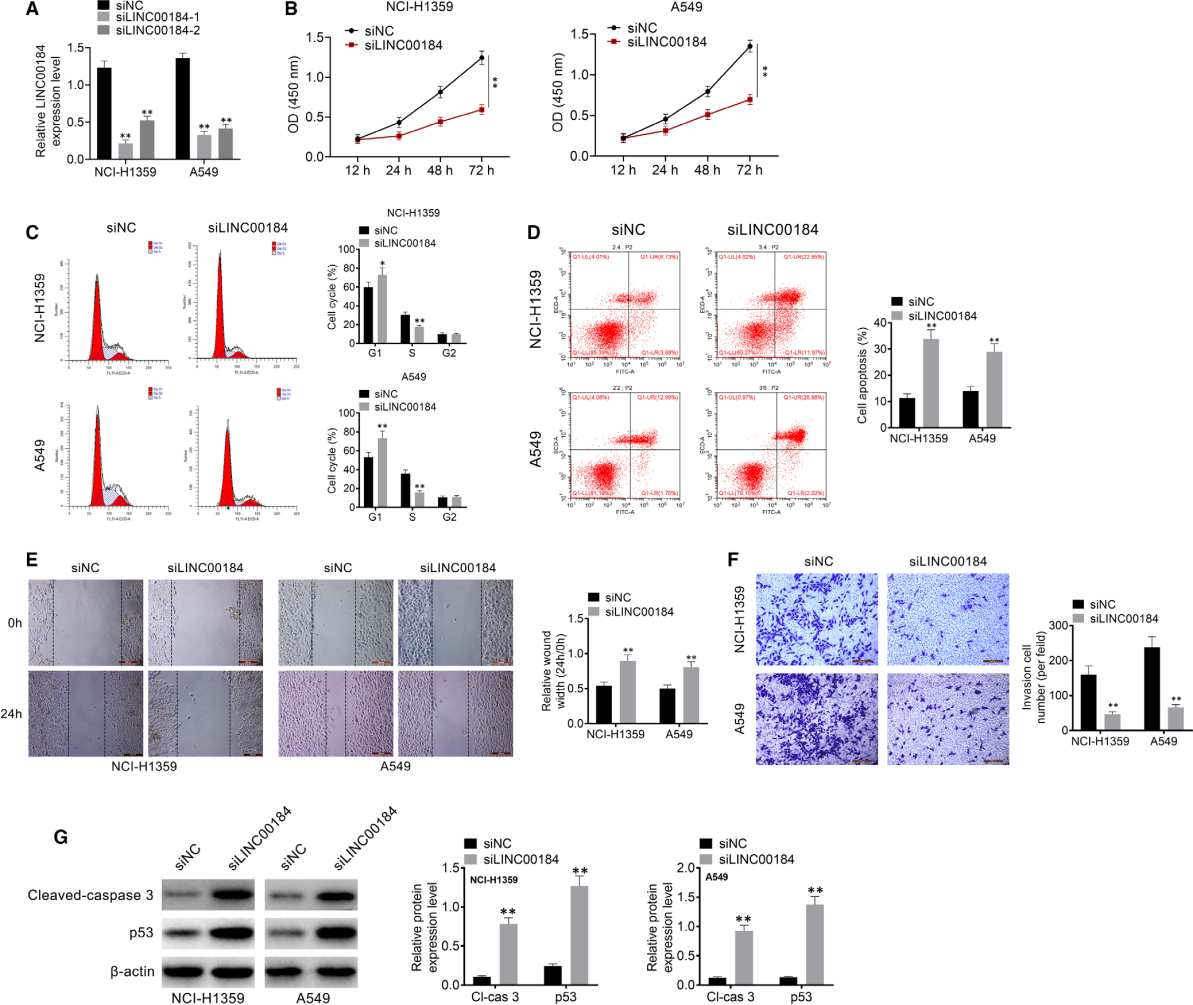
Knockdown of LINC00184 inhibited cell activities in NSCLC cell lines. A, NSCLC cell lines NCI‐H1359 and A549 were transfected with siLINC00184‐1, siLINC00184‐2 or siNC, the knockdown efficiency was detected using qRT‐PCR. B, Cell proliferation was detected using CCK‐8 assay. C, Cell cycle and D, cell apoptosis were detected using flow cytometry. E, Cell migration was detected using wound healing assay. F, Cell invasion was measured using transwell assay. G, The expressions of cleaved‐caspase‐3 and p53 in NCI‐H1359 and A549 cells were examined by Western blot analysis. Data are shown as mean ± SD. *P < 0.05, **P < 0.01 vs siNC group

To investigate the effects of LINC00184 on cellular activities in NSCLC, the knockdown vectors of LINC00184 (siLINC00184‐1 and siLINC00184‐2) were transfected into NSCLC cell lines NCI‐H1359 and A549. siLINC00184‐1 displayed stronger knockdown efficiency (Figure 2A). Therefore, siLINC00184‐1 transfection was selected to carry out the following experiments. Data from the CCK‐8 assay showed that compared to siNC transfection, siLINC00184 transfection significantly inhibited cell proliferation in NCI‐H1359 and A549 cell lines (*P* < 0.01, Figure 2B). Cell cycle and apoptosis were measured using flow cytometry. As shown in Figure 2C, the cell cycle was arrested in the G1 phase after siLINC00184 transfection in NCI‐H1359 and A549 cells. Additionally, flow cytometry data showed that apoptosis was significantly increased in the siLINC00184 group compared with that in the siNC group (*P* < 0.01, Figure 2D). The wound healing assay was utilized to assess cell migration activity (Figure 2E); results showed that the relative wound width of the siLINC00184 groups was significantly greater than that of the siNC groups (*P* < 0.01). Moreover, cell invasion activities were analysed using the transwell assay. The number of invasive cells in the siLINC00184 groups was lower than that in the siNC groups in NCI‐H1359 and A549 cell lines (*P* < 0.01, Figure 2F). The expression levels of cleaved caspase‐3 and p53 in NCI‐H1359 and A549 cells were examined by Western blot analysis. As shown in Figure 2G, siLINC00184 significantly increased the expression of cleaved caspase‐3 and p53 in NCI‐H1359 and A549 cells compared with that in cells transfected with siNC (*P* < 0.01). These data confirmed that LINC00184 knockdown repressed cell proliferation, migration and invasion, blocked the cell cycle and promoted apoptosis in NSCLC cell lines.

### 3.3 LINC00184 directly targeted miR‐524‐5p in NSCLC

**FIGURE 3:**
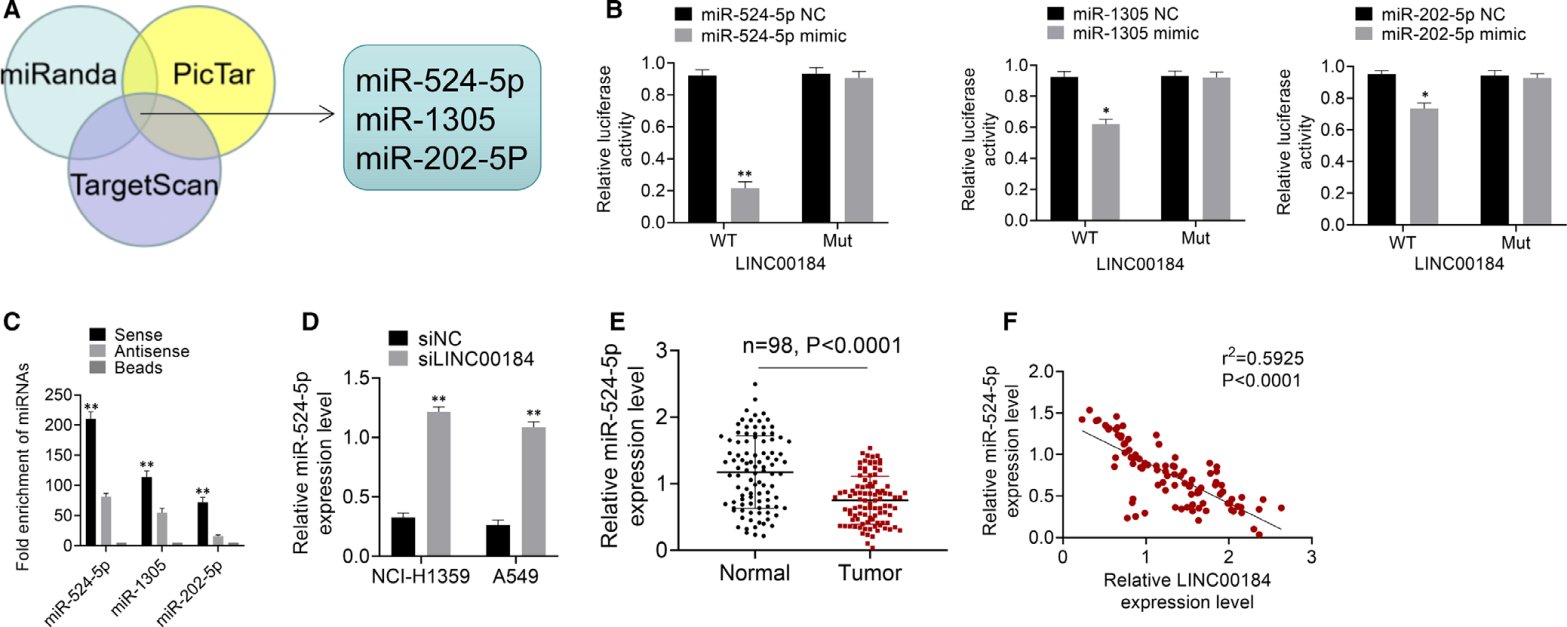
LINC00184 directly targeted miR‐524‐5p in NSCLC. A, The target miRNAs of LINC00184 was predicted using PicTar, miRanda and TargetScan. B, The relative miRNA enrichment was detected using RNA pull‐down assay. C, The relative luciferase activities were measured using luciferase reporter assay. D, The expression level of miR‐524‐5p in NSCLC cell lines was detected using qRT‐PCR. E, The expression level of miR‐524‐5p in 98 paired of NSCLC tumour tissues and normal tissues were detected using qRT‐PCR. F, The relationship between LINC00184 expression and miR‐524‐5p expression was analysed by Pearson's chi‐square test. Data are shown as mean ± SD. *P < 0.05, **P < 0.01 vs miR‐NC groups, NC groups, siNC groups or normal groups

MiR‐524‐5p, miR‐1305 and miR‐202‐5p were predicted to be the target miRNAs of LINC00184 by PicTar, miRanda and TargetScan (Figure 3A). The prediction was then verified through the RNA pull‐down and luciferase reporter assays. As shown in Figure 3B, the miR‐524‐5p group exhibited the maximum relative luciferase activity. Similarly, data from the RNA pull‐down assay suggested that LINC00184 directly targeted miR‐524‐5p (Figure 3C). In NSCLC cell lines, the expression level of miR‐524‐5p was significantly enhanced by LINC00184 knockdown (*P* < 0.01, Figure 3D). Moreover, miR‐524‐5p was significantly down‐regulated in NSCLC tumour tissues when compared to that in normal tissues (*P* < 0.0001, Figure 3E). Additionally, the expression level of LINC00184 in NSCLC tumour tissues was negatively correlated with miR‐524‐5p expression (Figure 3F). These results illustrated LINC00184 sponge binding to miR‐524‐5p in NSCLC.

### 3.4 LINC00184/miR‐524‐5p directly targeted HMGB2 in NSCLC

**FIGURE 4:**
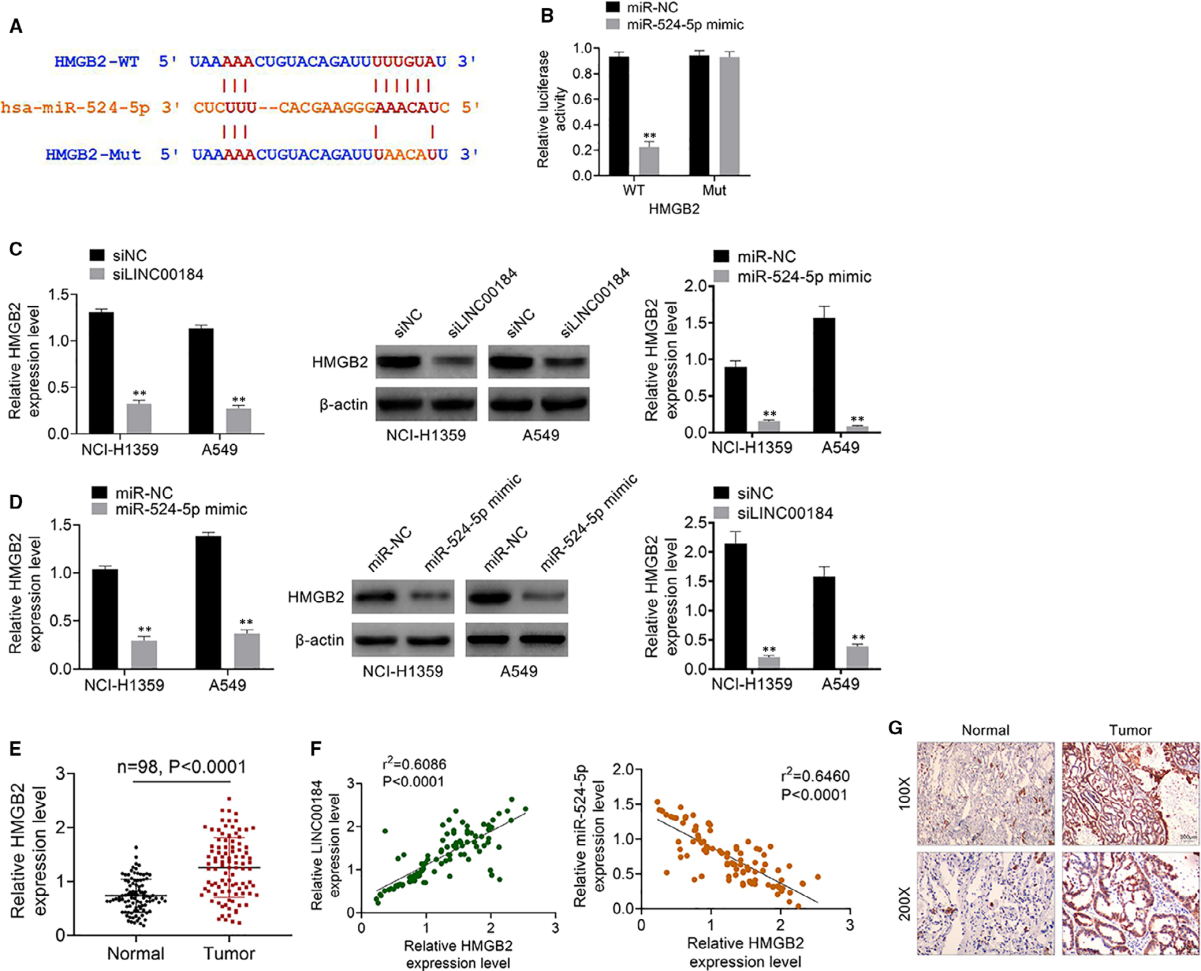
LINC00184/miR‐524‐5p directly targeted HMGB2 in NSCLC. A, The target downstream gene of miR‐524‐5p was predicted using TargerScan. B, The relative luciferase activities were measured using luciferase reporter assay. (C,D) The expression level of HMGB2 in NSCLC cell lines was detected using qRT‐PCR and Western blot. E, The expression level of HMGB2 in 98 paired of NSCLC tumour tissues and normal tissues were detected using qRT‐PCR. F, The relationship between HMGB2 expression and LINC00184/miR‐524‐5p expression was analysed by Pearson's chi‐square test. G, The HMGB2 expression in NSCLC tumour tissues and normal tissues was detected by IHC. Data are shown as mean ± SD. *P < 0.05, **P < 0.01 vs miR‐NC groups, siNC groups or normal groups

We predicted that *HMGB2* was the downstream gene of miR‐524‐5p by TargetScan; its binding sites are shown in Figure 4A. Luciferase reporter assay verified this prediction (Figure 4B). Data from qRT‐PCR and Western blot analysis showed that the expression of HMGB2 was significantly decreased after siLINC00184 or miR‐524‐5p mimic transfections in NCI‐H1359 and A549 cell lines (*P* < 0.01, Figure 4C,D). The expression levels of HMGB2 in NSCLC tumour and normal tissues were detected via qRT‐PCR; results showed that HMGB2 expression was significantly up‐regulated in tumour tissues compared with that in the normal group (*P* < 0.0001, Figure 4E). HMGB2 expression was positively correlated with LINC00184 expression and negatively correlated with miR‐524‐5p expression in NSCLC tumour tissues (Figure 4F). Additionally, IHC results revealed that HMGB2 was highly expressed in NSCLC tumour tissues (Figure 4G). These results indicate that LINC00184/miR‐524‐5p directly targeted HMGB2 in NSCLC.

### 3.5 LINC00184 regulated cell activities by targeting miR‐524‐5p/HMGB2 in NSCLC cell lines

**FIGURE 5:**
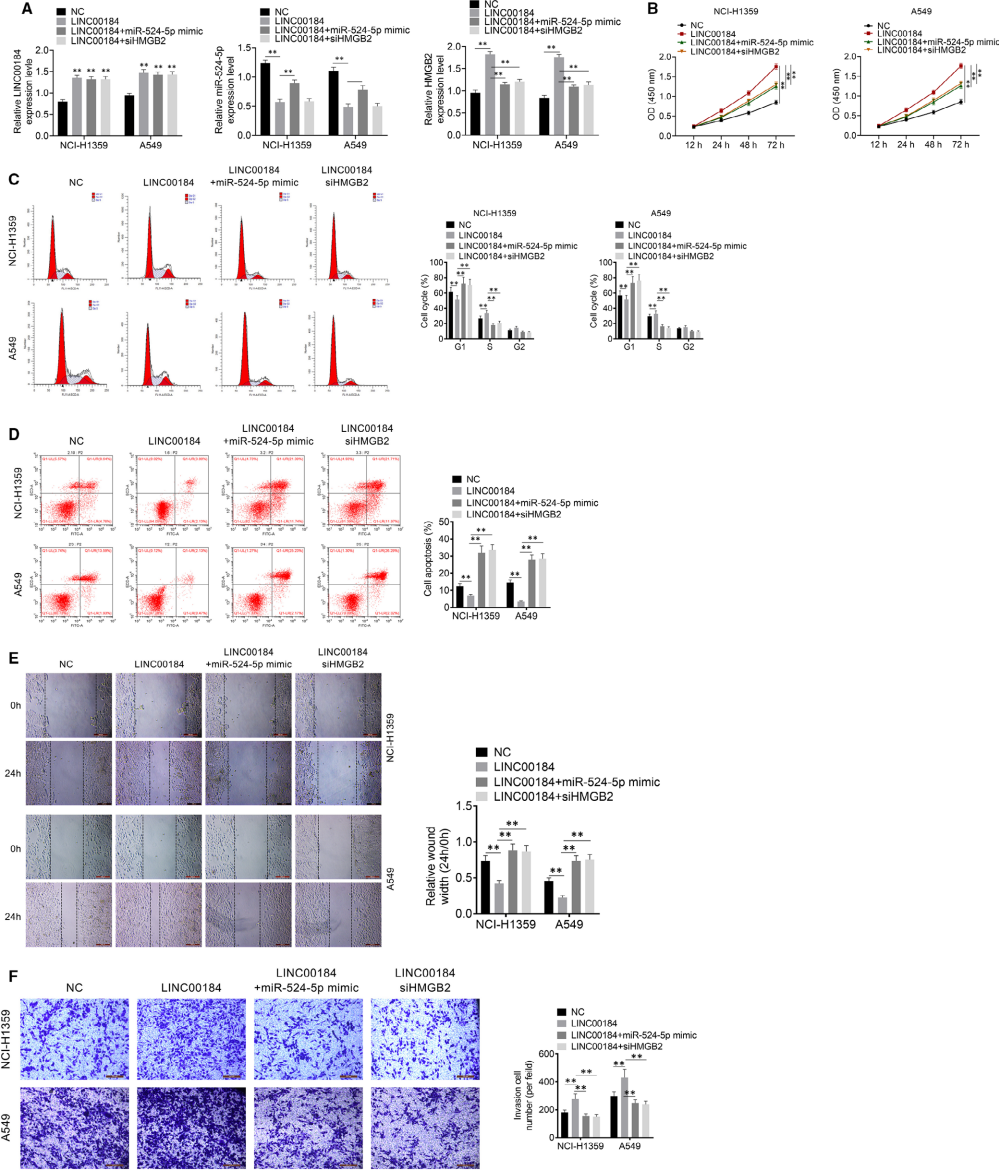
LINC00184 effects on NSCLC cell activities by targeting miR‐524‐5p/HMGB2. A, The expressions of LINC00184, miR‐524‐5p and HMGB2 were detected using qRT‐PCR. B, CCK‐8 was performed to measure cell proliferation. C, Cell cycle and D, cell apoptosis were both assessed using flow cytometry. E, Cell migration was detected using wound healing assay. F, Cell invasion was detected using transwell assay. Data are shown as mean ± SD. **P < 0.01 vs NC group, ^##^ *P* < 0.01 vs LINC00184 group

To further investigate the potential mechanism of action of LINC00184 in NSCLC, NCI‐H1359 and A549 cell lines were divided into four groups and transfected with negative control (NC), LINC00184 (LINC00184 overexpressed plasmid), LINC00184 + miR‐524‐5p mimic and LINC00184 + siHMGB2, respectively. The expression levels of LINC00184, miR‐524‐5p and HMGB2 were identified by qRT‐PCR analysis (Figure 5A). Data from the CCK‐8 assay showed that LINC00184 up‐regulation promoted cell proliferation, whereas miR‐524‐5p mimic or siHMGB2 treatments suppressed cell proliferation caused by LINC00184 transfection (Figure 5B). As shown in Figure 5C, LINC00184 transfection promoted the G1 phase, whereas miR‐524‐5p mimic or siHMGB2 transfections arrested the G1 phase in NCI‐H1359 and A549 cell lines. Additionally, the overexpression of LINC00184 significantly reduced apoptosis, while the overexpression of miR‐524‐5p and HMGB2 knockdown enhanced apoptosis (*P* < 0.01, Figure 5D). Moreover, results from the wound healing and transwell assays showed that LINC00184 transfection increased cell migration and invasion activities, whereas miR‐524‐5p or siHMGB2 transfections inhibited these activities (Figure 5E,F). These data elucidated that LINC00184 affected cell cycle, proliferation, apoptosis, migration and invasion via modulating the miR‐524‐5p/HMGB2 axis.

### 3.6 Knockdown of LINC00184 inhibited NSCLC tumour growth in vivo

**FIGURE 6:**
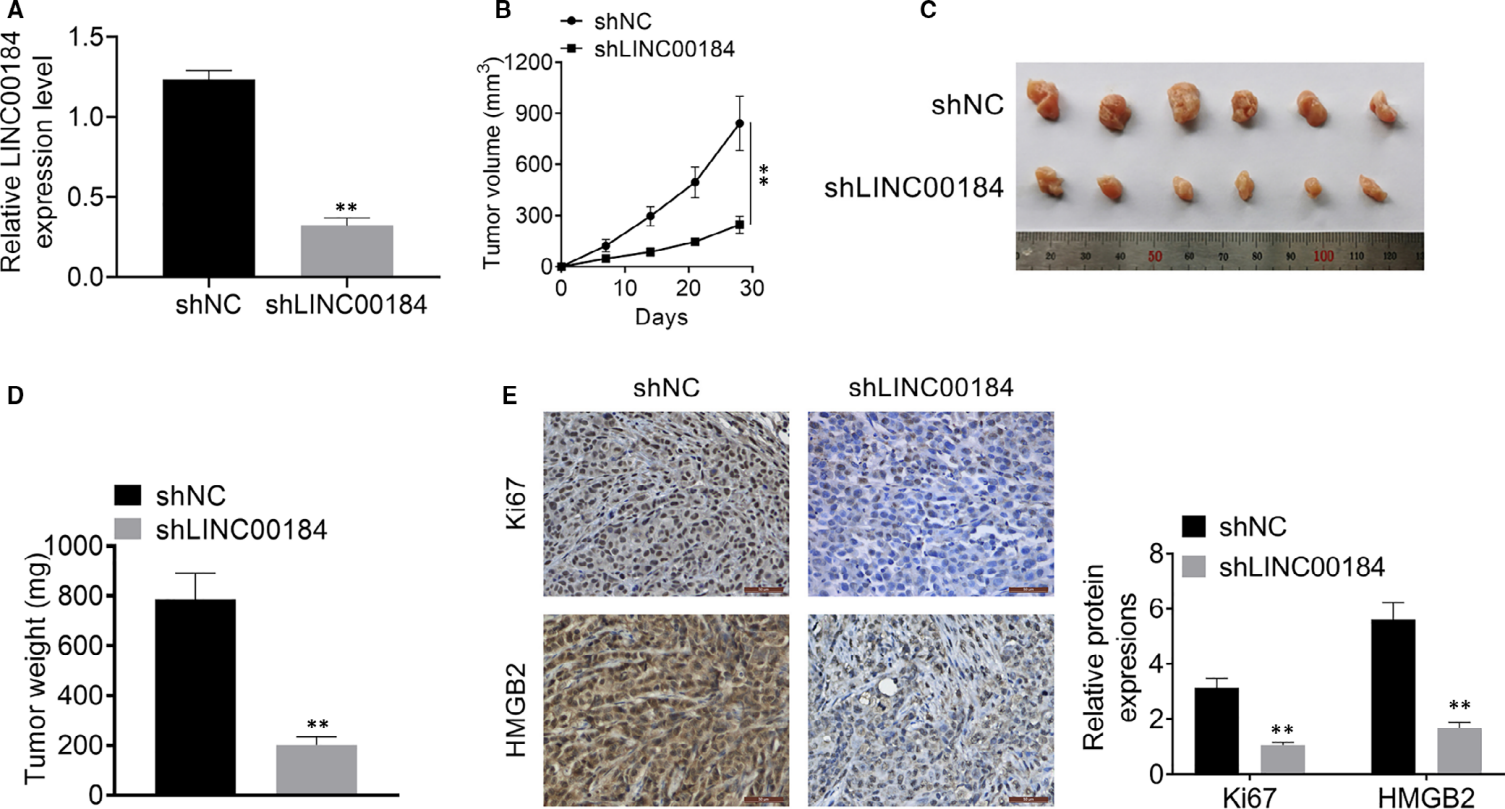
Knockdown of LINC00184 inhibited NSCLC tumour growth and proliferation in vivo. A, The knockdown efficiency of shLINC00184 transfection waas detected using qRT‐PCR. B, The tumour volume of NSCLC mice model was assesed weekly. C, The tumour size of NSCLC mice model was assesed after the mice were killed. D, The tumour weight of NSCLC mice model was assesed after the mice were killed. E, The Ki67 and HMGB2 expressions were detected using IHC. Data are shown as mean ± SD. **P < 0.01 vs shNC group

To examine the effects of LINC00184 on NSCLC in vivo, NSCLC nude mouse models were established and injected with A549 cells transfected with shLINC00184/shNC. LINC00184 expression in the shLINC00184 group was significantly suppressed when compared to that in the shNC group (*P* < 0.01, Figure 6A). LINC00184 knockdown significantly inhibited tumour volume, weight and growth in vivo (*P* < 0.01, Figure 6B,C,D). Data from IHC showed that the expression of HMGB2 and Ki67 was repressed in the shLINC00184 group compared with that in the shNC group (Figure 6E). These results proved that the down‐regulation of LINC00184 inhibited NSCLC tumour growth and proliferation in vivo.

## 4 DISCUSSION

Lung cancer presents the highest incidence and mortality rates among various malignant tumours in the world. NSCLC is the most common type of LC.^4^ Although great progress has been made in early screening, minimally invasive treatment technology and biologically targeted treatment, the 5‐year survival rate of NSCLC remains low.^5^ A variety of studies show that the development of tumours is often accompanied by genetic mutation and abnormal protein expression. A thorough understanding of the molecular mechanism underlying the occurrence and development of NSCLC is conducive to the evolution of effective targeted treatment measures and identification of clues for the early diagnosis of NSCLC.

In recent years, increasing attention has been paid to the role of lncRNAs in NSCLC. Xia et al^18^ reported that lncRNA PLCA2 suppressed cell migration and invasion and negatively modulated miR‐21 expression in NSCLC. Zhang et al^19^ showed that lncRNA NR2F2‐AS1 accelerated cell viability via binding to BMI1, which regulated miR‐302b expression in NSCLC. Miao et al^20^ found that lncRNA HAND2‐AS1 effected NSCLC cell stemness, migration and invasion by modulating transforming growth factor‐β1 (TGF‐β1). However, the role of LINC00184 in NSCLC has not been reported. The mechanism of action of LINC00184 in tumours is scarcely reported, except in oesophageal cancer.^16^ Therefore, the role of LINC00184 in cancer should be the focus of future research.

In the current study, we confirmed that LINC00184 was up‐regulated in NSCLC tumour tissues and cell lines and its high expression in NSCLC tissues indicated a poor prognosis for NSCLC patients. LINC00184 expression in tumour tissues was positively correlated with the clinical stages of NSCLC. LINC00184 expression was down‐regulated by siLINC00184 transfection in NSCLC cell lines NCI‐H1359 and A549. Results of the CCK‐8 assay, used to detect cell proliferation, showed that siLINC00184 transfection inhibited cell proliferation in both cell lines. Flow cytometry data showed that LINC00184 knockdown blocked the cell lines in the G1 phase and facilitated apoptosis. Moreover, siLINC00184 transfection suppressed cell migration and invasion in human NSCLC cell lines.

MiR‐524‐5p was reported to be down‐regulated in several tumours such as gastric cancer,^21^ glioblastoma,^22^ and oral squamous cell carcinoma.^14^ HMGB2 was reported to play a role in several cancers, including colorectal carcinoma,^23^ gastric,^24^ and pancreatic cancer.^25^ However, the function and effects of miR‐524‐5p and HMGB2 in NSCLC were unclear. In the study, we predicted LINC00184 binding to miR‐524‐5p using online bioinformatics tools, which was verified by luciferase reporter and RNA pull‐down assays. TargetScan and luciferase reporter assays were performed to predict and verify that HMGB2 was the downstream gene of miR‐524‐5p/LINC00184. Additionally, we found that miR‐524‐5p was down‐regulated and HMGB2 was up‐regulated in NSCLC tumour tissues. LINC00184 expression was positively correlated with HMGB2 expression and negatively correlated with miR‐524‐5p expression. Moreover, we confirmed that LINC00184 affects cellular activities by regulating miR‐524‐5p/HMGB2 through a rescue experiment. Finally, we showed that LINC00184 knockdown could inhibit tumour growth and proliferation in vivo.

In conclusion, our study demonstrated that LINC00184 suppressed cell migration, invasion and proliferation, arrested the cell cycle and promoted apoptosis by targeting the miR‐524‐5p/HMGB2 axis. These results provide novel insights and suggest potential molecular targets for NSCLC treatment.

## CONFLICT OF INTEREST

The authors declare that they have no competing interests.

## AUTHOR CONTRIBUTIONS

**Wuming Wang:** Conceptualization (equal); Data curation (equal); Investigation (equal); Methodology (equal); Resources (equal); Software (equal); Writing‐original draft (equal). **Lin Li:** Conceptualization (equal); Data curation (equal); Formal analysis (equal); Investigation (equal); Methodology (equal); Resources (equal); Writing‐original draft (equal). **Long Zhao:** Conceptualization (equal); Investigation (equal); Methodology (equal); Validation (equal); Visualization (equal); Writing‐original draft (equal); Writing‐review & editing (equal).

## Data Availability

All data generated and/or analysed during this study are included in this published article.
